# Identification of Mustard Aldehyde Dehydrogenase (ALDH) Gene Family and Expression Analysis Under Salt and Drought Stress

**DOI:** 10.3390/genes16050559

**Published:** 2025-05-07

**Authors:** Yuling Zheng, Shanshan Wang, Ling He, Rui Chen, Wei Zhang, Huachuan He, Hanbing Hu, Xiaoyun Liu, Heping Wan, Chunhong Wu

**Affiliations:** Hubei Engineering Research Center for Protection and Utilization of Special Biological Resources in the Hanjiang River Basin, College of Life Science, Jianghan University, Wuhan 430056, Chinawanheping@jhun.edu.cn (H.W.)

**Keywords:** *ALDH* gene family, *Brassica juncea*, drought stress, plant stress resistance, salt stress

## Abstract

Background/Objectives: Abiotic stresses severely constrain the yield of *Brassica juncea,* and aldehyde dehydrogenases (ALDHs) play a pivotal role in plant stress resistance. This study aims to systematically identify the *ALDH* gene family members in *B. juncea* and elucidate their expression patterns under salt and drought stress. Methods: Using the Arabidopsis thaliana AtALDH proteins as seed sequences, BLASTp alignment was performed against the *B. juncea* whole-protein sequence database, combined with the conserved domain PF00171 of the ALDH proteins. A total of 39 *BjALDH* gene family members were identified, and their physicochemical properties, structures, phylogenetic relationships, interspecies collinearity, and intraspecies collinearity were analyzed. The qRT-PCR method was employed to quantify the relative expression levels of the *BjALDH* genes potentially associated with stress resistance under various treatments, and their effects on drought and salt stress tolerance were evaluated. Results: The results demonstrated that *BjALDH* were universally significantly upregulated under salt stress, while exhibiting predominantly upregulated trends under drought stress. These findings suggest that *BjALDH* may enhance plant resistance to both salt and drought stress by modulating the aldehyde metabolic pathways. Conclusions: This study provides a theoretical basis for elucidating the functional roles and molecular genetic mechanisms of the *BjALDH* gene family in *B. juncea* under salt and drought stress.

## 1. Introduction

Mustard (*Brassica juncea*) is formed by the natural compound hybrid of cabbage (*Brassica rapa*, AA) and *Brassica nigra* (BB) [1,2]. It is one of the most important part of the whole world’s oil crops, and plays an important role in the field of agricultural production and food safety. It is widely planted worldwide due to its rapid growth, high yield, and strong adaptability [3,4]. Under field conditions, mustard is often subjected to abiotic stresses, such as drought, high temperature, low temperature, salinity, and heavy metals, and these adverse environmental factors have always seriously affected its yield and quality [5]. Therefore, it is important to study the tolerance mechanism of mustard under abiotic stress to improve its stress resistance and promote its high yield and quality.

Salt stress is a relatively common abiotic stress factor, which has a great impact on crop growth and yield, and causes serious economic losses. Excessive salinity in the soil has caused a series of environmental problems, with salinity affecting more than 6% of the world’s total land area (about 800 million hectares) [6]. With the increasing occurrence of extreme weather, improper irrigation, misuse of chemical fertilizers, and industrial emissions, the global ecological environment continues to deteriorate, soil salinity gradually increases, and soil salinization becomes more and more serious [7], bringing great environmental challenges to agricultural production [8]

Drought stress has an inhibitory effect on cell division, leading to the increase of cell membrane permeability to produce excessive free radicals, destroying the metabolic balance of reactive oxygen species, aggravating the degree of membrane lipid peroxidation, resulting in the inactivation of various enzymes in cells, and thus damaging the structure and function of the membrane [9,10]. In addition, drought stress also leads to cell dehydration, reduced cell volume, increased solute concentration, and viscosity, resulting in protein aggregation, leading to inhibition of photosynthetic enzyme activity, destruction of photosynthetic tissue, and plant growth restriction, and even death [11].

Aldehyde dehydrogenases (ALDHs) are enzymes that rely on NAD^+^/NADP^+^ as a cofactor to irreversibly convert endogenous and exogenous aromatic/aliphatic aldehydes into corresponding carboxylic acids, thereby reducing the toxic effects of aldehydes [12,13], and are able to adapt to environmental changes by maintaining aldehyde homeostasis. In addition, it takes part in and fulfils a momentous role in physiological processes, such as carnitine biosynthesis, glycolysis, gluconeogenesis, and amino acid metabolism [14]. In a study of soybeans, Kotchoni et al. [15] found that the soybean genome contains 18 unique *ALDH* sequences, encoding five members of the *ALDH* family involved in a wide range of metabolic and molecular detoxification routes. Yang et al. [16] found that 17 *ALDH* genes were distributed on nine chromosomes of the melon genome and were divided into 10 different subgroups by phylogenetic analysis. Furthermore, the expression patterns of the *ALDH* gene family under six stress types were salt, low temperature, waterlogging, powdery mildew, Fusarium blight, and glutinous stem blight. The results showed that *CmALDH2C4* and *CmALDH11A3* were significantly expressed under all six stresses, indicating that these two *CmALDH* genes play key roles in the stress response of melon. Bhuya et al. [17] found that most of the *CaALDH* genes in pepper were highly responsive to salt stress, and their expression level was significantly upregulated. Kirch et al. [18] found that upregulation of *AtALDH3I1* and *AtALDH7B4* genes in Arabidopsis enhanced tolerance to the osmotic and oxidative stressors in Arabidopsis. Islam et al. [19] conducted a genome-wide analysis of the potato and identified a total of 22 *ALDH* genes, with 50% *StALDHs* observed, with *StALDH18A2* showing the highest upregulation, and 62.5% *StALDHs*, including *StALDH10A2* and *StALDH18A2*.

Recently, the *ALDH* gene family has been characterized and extensively studied in a variety of plant species, including Arabidopsis, soybean, grape, sorghum, potato, melon, and poplar [13,15,16,18,19,20]. However, there are still no studies on the *ALDH* gene family in *B. juncea*, and this study fills a gap in this field. In this study, based on the genome information of mustard, we identified the members of the *ALDH* gene family, analyzed them, and analyzed the expression difference of the *BjALDH* genes under salt treatment and drought treatment, and further explored the specific biological function of the *BjALDH* gene family members. It aims to provide an important theoretical basis and germplasm resources for obtaining high quality anti-resistant mustard varieties, and to elucidate the potential roles of the *ALDH* gene family in the adaptation to drought and salt stress of *B. juncea*.

## 2. Materials and Methods

### 2.1. Materials and Their Treatment

The mustard resource 22-438-2 provided by Professor Wan Zhengjie from the mustard breeding research team of Huazhong Agricultural University was selected as the material for this study. This variety has strong resistance to abiotic stress, such as salt tolerance (unpublished identification results). The selected mustard strain (22-438-2), provided by the Chinese national modern agriculture (special vegetables) industrial technology system, has a strong salt tolerance. The stress levels, 180 mM NaCl for salt stress and 300 mM mannitol for drought stress, are selected for the best observation and analysis of gene expression changes which were proved by preliminary experiments. The mustard seeds were sown into a tray with a Hoagland nutrient solution (32.5 cm × 24.5 cm × 4.5 cm), and sterile gauze was spread on the plate to form a seeding cell. The seeds were treated under the temperature of 20 °C, 65% humidity, and long sunshine (18 h light and 6 h dark cycle). After 14 days of sowing and growth of the mustard seeds, 5 mustard seedlings with good growth were transferred to NaCl (180 mM) and mannitol (300 mM) as the experimental group, and 5 well-growing mustard seedlings were transferred to the Hoagland nutrient solution without NaCl and mannitol as the control group. The treatment adopted was set for a duration of 12 h, and the materials were sampled, flash-frozen, and stored at −80 °C for subsequent qRT-PCR experiments. The experiment was conducted in the greenhouse of the College of Life Sciences, Jianghan University, Wuhan City, Hubei Province, China (30°30′ N, 114°9′ E).

### 2.2. Identification of the BjALDH Gene Family Members and Chromosome Localization

Obtain the genome sequence files and protein sequence files from the Ensembl Plants database (https://plants.ensembl.org/index.html, accessed on 11 August 2024), the genome sequence files and protein sequence files from the tea tree (*C. sinensis*) genome database (http://tpia.teaplants.cn/, accessed on 5 September 2024), the Arabidopsis ALDH protein sequences were obtained as seed sequences from the Arabidopsis (*A. thaliana*) genome database (TAIR: https://www.arabidopsis.org/, accessed on 5 September 2024). These sequences were used for the BLASTp search with a threshold set at E < 10^−10^ to identify potential BjALDH proteins in the mustard whole protein sequence. The hidden Markov model (HMM) file for the conserved domain of ALDH PF00171 was downloaded from the Pfam database (http://pfam.xfam.org/, accessed on 6 September 2024) [21].

The mustard protein sequences containing this domain were screened by an HMMER 3.1 software (http://www.hmmer.org/, accessed on 7 September 2024) search. The members of the *ALDH* families obtained by the two methods were crossed, redundant sequences were removed, and the *ALDH* gene domain was further determined by the NCBI-CDD (https://www.ncbi.nlm.nih.gov/cdd/, accessed on 20 September 2024) and Pfam database to obtain the *ALDH* gene, and the name of the *BjALDH* gene was named *BjALDH1*~*BjALDH39*. Using the Expasy ProtParam (https://web.expasy.org/protparam/, accessed on 10 October 2024) online tool to predict biochemical parameters, such as the physical and chemical properties of the BjALDH protein, the predicted structure was implemented by the Plant-mPLoc website [22]. The BjALDH protein secondary structure was predicted using the SMOPA online tool [23]. Using the downloaded annotation files to retrieve the chromosomal location information of *BjALDH* and Visualizing it with TBTools v2.225 [24].

### 2.3. Phylogenetic and Evolutionary Analysis of the ALDH Proteins in Mustard

The AtALDH protein sequence of Arabidopsis was downloaded from TAIR. The phylogenetic tree was constructed in MEGA 11 software with the adjacency method (Neighbor joining, NJ), the boot-trap parameter was set to 1000 times, and the phylogenetic tree was beautified with the online website iTOL (https://itol.embl.de/, accessed on 5 September 2024) [25,26]. To explore the phylogenetic relationship between the BjALDH proteins, a phylogenetic tree for Arabidopsis (14), tea tree (27), and mustard was constructed using NJ with 1000 bootstrap repeats in MEGA 11 software (Supplementary File S2).

### 2.4. Gene Structure and Protein Conserved Domain Analysis of BjALDH

The gene structure of the *ALDH* family members was obtained based on the genome annotation information. Conserved domains of the BjALDH proteins were obtained from the NCBI-CDD website and the Pfam database. Their conserved motifs were obtained using the MEME 5.5.7 website (http://meme-suite.org/tools/meme, accessed on 12 October 2024) [27]. The cis-acting elements of 2000 bp upstream of the *BjALDH* gene were predicted via the Plant CARE website [28]. All data information visualization was performed using TBTools.

Using MCScanX2.0.0 software, the collinear relationships between the mustard, Arabidopsis, and tea tree *ALDH*, and among genes within the *BjALDH* genome, were analyzed by MCScanX [29]. Collinearity information visualization was performed using TBTools.

### 2.5. Analyzed by qRT-PCR Analysis of the ALDH Expression Pattern

A total RNA extraction kit (FastPure Universal Plant Total RNA Isolation Kit, Norozan, Nanjing, China) was used to extract the total RNA from the mustard samples. The cDNA was obtained by reverse transcription of the HiScript^®^ II Q Select RT SuperMix with gDNA wiper (Nanjing, Nanjing, China). The qRT-PCR primers were designed with the Primer 6.0 software (Table 1), and underwent qRT-PCR analysis with the AriaMx real-time PCR system (Agilent Technologies, Chaoyang, Beijing, China). The total volume of the qRT-PCR reaction system was 20 μL, consisting of 10 μL of 2 × PerfectStart^®^ Green qPCR SuperMix, 0.4 μL of upstream primers (Primer concentration: 10 μM; final concentration: 0.2 μM), 0.4 μL of downstream primers (Primer concentration: 10 μM; final concentration: 0.2 μM), 1 μL of cDNA template (200 ng/μL), and 8.2 μL of nucleic acid-free water. The qRT-PCR reaction steps were as follows: 95 °C pre-denaturation for 30 s; 95 °C denaturation for 5 s, 55 °C annealing for 1 min, 40 cycles. The internal label is the GAPDHF. Relative gene expression was calculated by the 2^−ΔΔCT^ method [30]. Three biological replicates were used for each sample. 

## 3. Results

### 3.1. Identification and Chromosome Mapping of Gene Families

**Figure 1:**
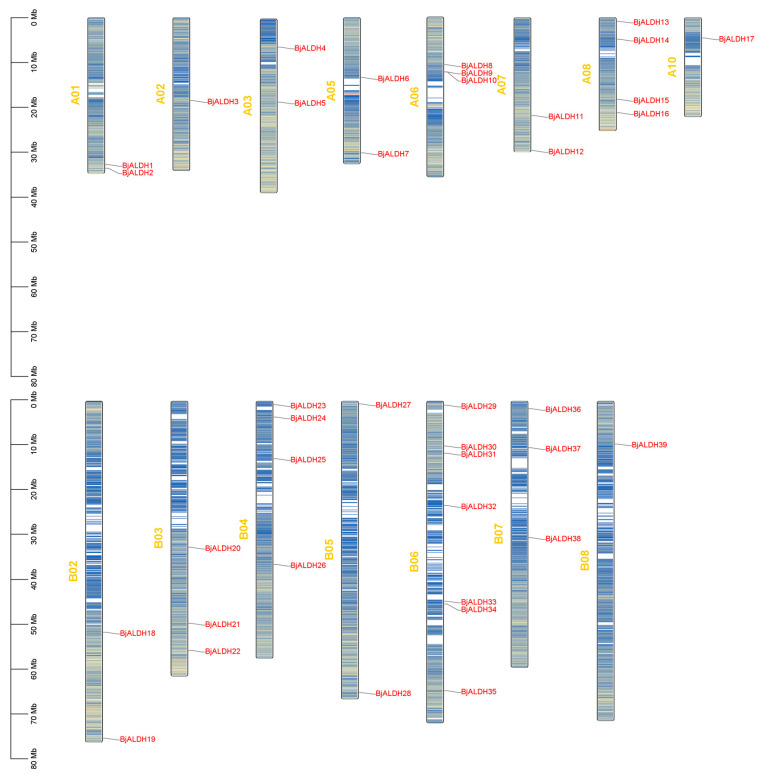
The arrangement of *BjALDH* across the chromosomes of *B. juncea*. Each chromosome name is indicated on the left side of the respective blue bar, with gene names presented on the right. The scale on the far left denotes the physical position in megabases (Mb).

Identification of the *BjALDH* conserved domains using NCBI-CDD and Pfam tools finally identified the 39 *BjALDH* family members, which were named *BjALDH1*~*BjALDH39* according to the order of their position on the chromosome, and located on 15 chromosomes. Of these, one *BjALDH* was on chromosomes A02, A10, and B08, two were on chromosomes A01, A03, A05, A07, B02, and B05, three were on chromosomes A06 and B03, four were on chromosomes A08 and B04, and seven were on chromosome B06 (Figure 1).

### 3.2. Physicochemical Properties and Subcellular Localization Prediction of the Proteins

Analysis of the physicochemical properties of the 39 *BjALDH* genes showed differences between the different BjALDH protein sequences (Table 2 and Supplementary File S1). The number of amino acids of the BjALDH protein is between 58 and 909, the molecular weight range is 5911.94 to 99,378.23 Da, the theoretical isoelectric point is 4.27 to 9.69, the instability index is between 22.42 and 44.8, the average hydrophobicity is between −0.206 and 0.066, indicating that the BjALDH protein may partially exhibit hydrophilicity.

### 3.3. Phylogenetic Analysis of the Proteins

**Figure 2:**
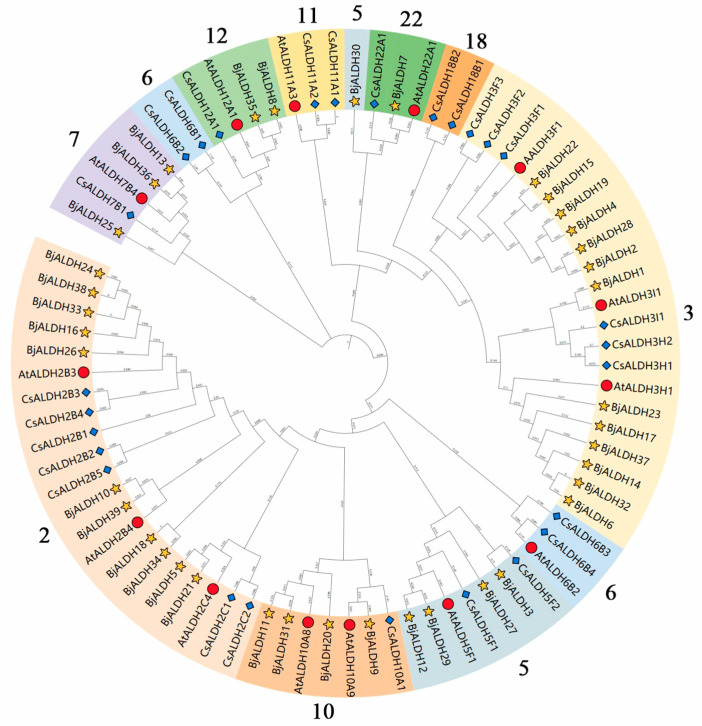
The phylogenetic tree of the ALDH proteins from *A. thaliana* (At), *C. sinensis* (Cs), and *B. juncea* (Bj). Different colors represent different subfamilies, numbers represent the numbering of each subfamilies, yellow stars represent *ALDHs* from Bj, red circles represent *ALDHs* from At, and blue diamonds represent *ALDHs* from Cs.

By phylogenetic analysis, the 80 ALDH proteins were divided into 10 families, including families 2, 3, 5, 6, 7, 10, 11, 12, 18, and 22 (Figure 2). In these three species, only tea trees had *ALDH18* family members, mustard plants had no *ALDH6* and *ALDH11* family members, and all three species were distributed in the other seven *ALDH* families. The number of family members for 2 and 3 is the highest in mustard and two other species, indicating that these two families may have more extensive and important biological functions in plants. By contrast, each species has only one family member for 22, implying that these families have functionally high conservation and specificity.

### 3.4. Analysis of Gene Structure, Conserved Motifs, and Promoter Cis-Acting Elements for BjALDH

**Figure 3:**
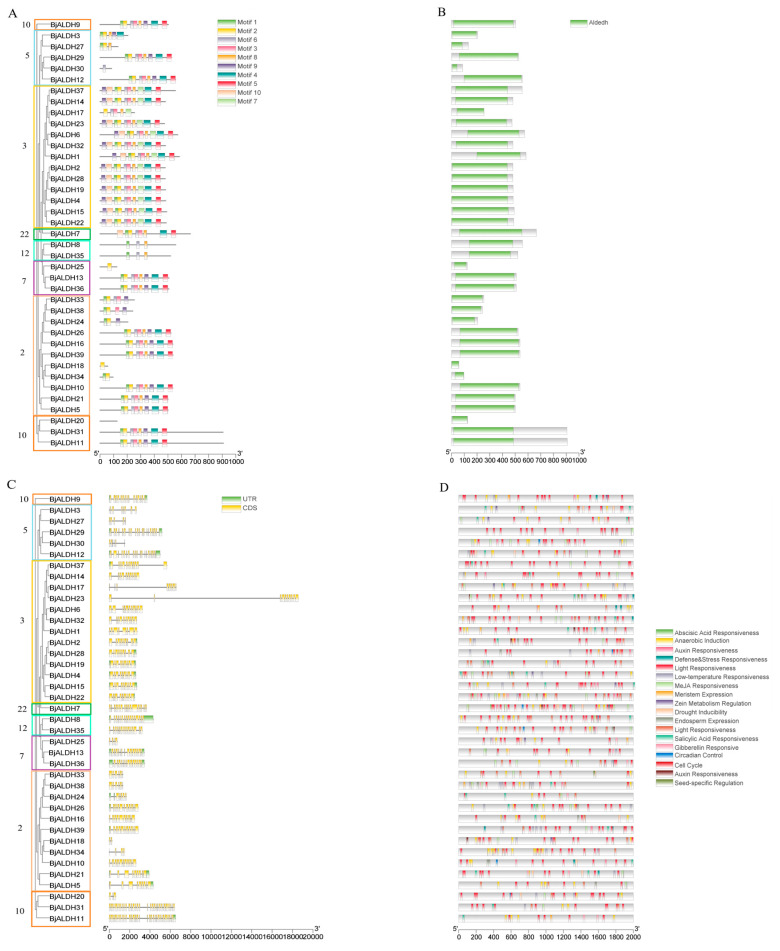
Conserved motifs of the *B. juncea ALDH* family (**A**), conserved domains (**B**), gene structure (**C**), and promoter cis-regulatory elements (**D**) analysis.

Analysis of the conserved motifs of the BjALDH proteins shows that Motif1, Motif2, Motif4 and Motif5 are present in most ALDH proteins, Motif9 in most families 2 and 3, and family 12 members contain Motif1, Motif6, and Motif8, with only one (Figure 3A). An analysis of the BjALDH protein showed that all proteins were found to contain the aldehyde domains (Figure 3B).

The results of the analysis of exon and intron structure (Figure 3C) show that the *BjALDHs* genes within the same family showed similarity in gene structure, with some obvious differences. In particular, the increase or loss of exons occurs in each family, which is particularly evident in family 2, where *BjALDH26* and *BjALDH39* are composed of 11 exons, while *BjALDH18* contains only 2 exons. Furthermore, although *BjALDH23* contains 10 exons, *BjALDH17* contains only 8 exons. The two genes also differ in length and structure.

An analysis of the promoter cis-acting elements suggests that *BjALDH* may have response mechanisms to stress, plant hormone signaling, and hypoxic environment, and this study identified multiple cis-acting elements, with up to 494 photoresponsive elements, far exceeding other cis-acting elements. Auxin and abscisic acid response are also abundant in the ALDH proteins. In addition, *BjALDH7*, with 49, contains the largest number of cis-acting elements, and it is speculated that it may play an important role in the growth, development, and response to various kinds of abiotic stress in mustard.

### 3.5. Collinear Analysis of the ALDH Gene Family

**Figure 4:**
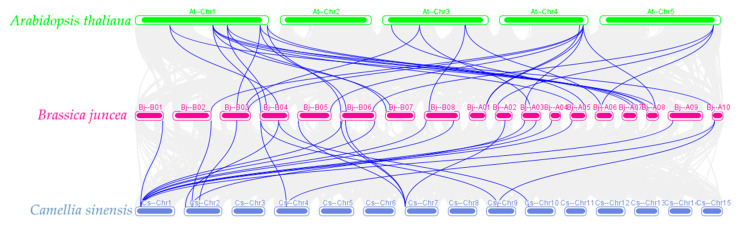
The collinearity relationship of *ALDH* genes between *A. thaliana*, *B. juncea*, and *C. sinensis*.

Mapping the collinearity of mustard (*B. juncea*), Arabidopsis (*A. thaliana*), and tea tree (*C. sinensis*), found that 29 co-linear gene pairs exist between *B. juncea* and *A. thaliana*, and 13 co-linear gene pairs exist between *B. juncea* and *C. sinensis* (*BjALDH19* and *CsALDH3F1*, *BjALDH20* and *CsALDH10A1*, *BjALDH26* and *CsALDH2B3*, *BjALDH26* and *CsALDH2B1*, *BjALDH23* and *CsALDH2H1*, *BjALDH23* and *CsALDH3H2*, *BjALDH27* and *CsALDH5F2*, *BjALDH29* and *CsALDH5F2*, *BjALDH30* and *CsALDH5F2*, *BjALDH3* and *CsALDH5F2*, *BjALDH4*, and *CsALDH3F1*, *BjALDH17*, and *CsALDH3H1*, *BjALDH17*, and *CsALDH3H2*) (Figure 4).

**Figure 5:**
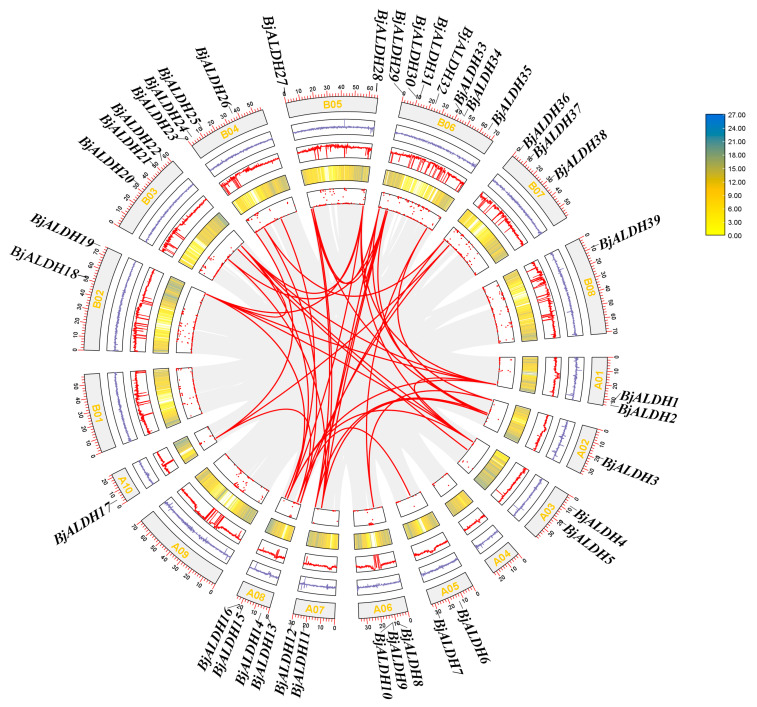
Synteny analysis among *BjALDH*. The circles in this diagram represent the unknown base ratio (N ratio), genetic density, GC ratio, GC skew, and chromosome length from the inside to the out. The names of the chromosomes are marked at the bottom of each chromosome, and indicate the location of *BjALDH*.

The results of the *BjALDH* collinearity analysis showed 37 pairs of collinity genes located in the species (Figure 5), such as *BjALDH2*–*BjALDH4*, *BjALDH8*–*BjALDH35*, *BjALDH11*–*BjALDH20*, and *BjALDH12*–*BjALDH27*.

### 3.6. Expression Pattern of BjALDH Under Drought and High Salt Conditions

**Figure 6:**
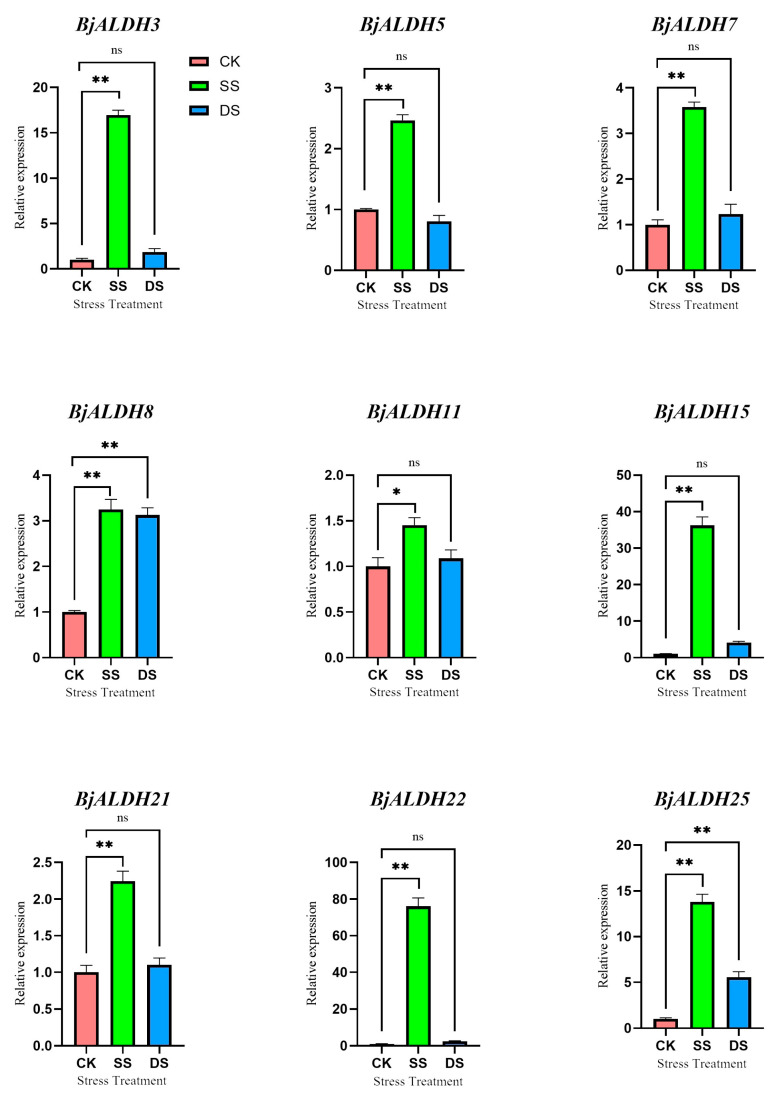
Non-biological stress-induced expression patterns of *BjALDH*. The x-axis represents various treatments of *B. juncea*. The y-axis indicates relative expression levels, calculated using the 2−∆∆Ct method. CK: normal; SS: salt stress; DS: drought stress; ** significant differences between treatments at *p* < 0.01; * significant differences between treatments at *p* < 0.05; ns: no significant difference. Data represent the mean ± SD for three biological replicates (n = 3).

In order to study the effect of *BjALDH* on mustard in drought and salt stress conditions, three hydroponic environments were selected: control (CK), simulating saline stress (SS: 180 mM) and drought stress (DS: mannitol, 300 mM). Salt stress conditions resulted in significant upregulation of *BjALDH3*, *BjALDH5*, *BjALDH7*, *BjALDH8*, *BjALDH11*, *BjALDH15*, *BjALDH21*, *BjALDH22*, and *BjALDH25*, especially *BjALDH22* by more than 40-fold compared with controls. While *BjALDH8* and *BjALDH25* were significantly upregulated under drought stress; *BjALDH3*, *BjALDH7*, *BjALDH11*, *BjALDH15*, *BjALDH21*, *BjALDH22,* and *BjALDH25* showed the trend of upregulation, but were not significantly different, possibly due to the fact that negative regulation or participation of metabolic pathways under drought conditions are not beneficial to plants under stress.(Figure 6) Overall, *BjALDH* showed an overall upregulation trend in response to drought/salt stress, implying that it may affect the physiological processes in response to drought/salt stress, thereby enhancing mustard resistance.

## 4. Discussion

Through the effective methods of gene family analysis, researchers can better, more comprehensively, and more easily understand the structure, function, and evolution of genes. In this study, the *ALDH* family was systematically analyzed in *B. juncea* for the first time, and its subfamily composition was significantly different from that of other species, suggesting species-specific functional differentiation. Plant *ALDH* is a key gene regulating the metabolic process of acetaldehyde. It existed early before the evolutionary separation of monocots and dicotyledons [17], which indicates that its evolutionary process is very long, and the potential genetic differentiation is very complex. Although the specific mechanism of the abiotic stress through acetaldehyde metabolism is not fully clear, the results show that it has a strong correlation with the process of stress, implying that it plays an important role in the relevant physiological changes of plants.

Related functions of *ALDH* in other plants have been widely reported, but have been rarely studied in mustard. In this study, BLASTp was used as the seed sequence in the mustard whole genome protein sequence database. Combined with HMM, CD-Search, and other genes to verify the domain integrity and reliability, 39 members of the *ALDH* gene family were finally identified, which were located on 15 chromosomes. The distribution patterns of *BjALDH* on chromosomes is significantly non-uniform (e.g., B06 chromosome enrichment). A similar situation occurred in *Callerya speciosa*, where *CsbHLH* was unevenly distributed on chromosomes, suggesting tandem replication events in the genome evolution of *C. speciosa* [31]. The *BjALDH* genes may also have a similar formation mechanism, and it is speculated that they are related to the differentiation of stress resistance functions based on the relationship between the *ALDH* gene family and plant stress resistance.

Through phylogenetic analysis of the protein sequences encoded by the *ALDH* genes in *A. thaliana*, *B. juncea*, and *C. sinensis*, we found that these proteins not only have numerous members, but form numerous evolutionary branches, significantly redundant with other families. This indicates that, in plants, the *ALDH* families not only appear early, but have a high degree of sequence divergence. The ancient origin of divergence and its diversification has led to reduced similarity between the different sequences, thus forming different clades in the phylogeny [32,33]. At the same time, the alienation and diversification of such evolutionary relationships also indicate that the biological functions of the *ALDH* family in plants also tend to be diversified [34].

This study found that the *BjALDH* proteins contain numerous light-responding elements and phytohormone signaling response elements that are usually associated with plant response to abiotic stress, especially drought stress and salinity stress [35]. For example, a considerable number of abscisic acid (ABA) response elements are contained in the *ALDH* proteins, which suggests that the *BjALDH* proteins may regulate stress response through the ABA signaling pathway, implying that the *ALDH* gene family may play an important role in these physiological activities. At the same time, we found that, among the 10 subfamilies, the family 2 and 3 proteins accounted for the highest proportion in mustard, 26.25% and 27.5%, respectively, while the 18 subfamily proteins accounted for the lowest proportion, only 2.5%, indicating that the 18 subfamilies are very conservative over the other subfamilies in terms of gene evolution, while possible gene family duplication events occurred more in other subfamilies. This property of the *ALDH* family has been demonstrated in previous studies [36,37]. An analysis of the *BjALDH* gene structure and the conserved domains suggests that it is evolutionary conserved among several plant species. By analysis of the *ALDH* gene family in mustard, Arabidopsis, and tea tree plants, 29 pairs were found between Arabidopsis and mustard, and 37 pairs were found between tea trees and mustard.

The role of the *ALDH* genes in plant response to stress has been widely reported in many plants. A study by Guo et al. [38] of low temperature stress in the potato *ALDH2B7a* gene four methylation status changes, and in the tobacco *StALDH2B7a* homologous gene expression, silent mutants appeared when the low temperature tolerance is weakened, providing preliminary evidence that *ALDH2B7a* is a positive regulation gene for low temperature stress. Subsequent studies have pointed out that *StALDH2B7a* in response to potato cold stress to remove reactive oxygen produced excess aldehydes. Gao and Han [39] found that rice (*Oryza sativa* L.) *ALDH* genes responded significantly to drought and salt stress, with five *ALDHs* upregulated during both drought and high salt stress. Conversely, three *ALDHs* were downregulated during drought, and only *OsALDH11*, *OsALDH22*, and *OsALDH18-1* were downregulated during salt stress. These results suggest that some rice *ALDH* genes may have a potential role in the response and tolerance of abiotic stresses (such as drought and salt). Correspondingly, the expression data under stress conditions in this study showed the significant difference between expression patterns of different members of the mustard *ALDH* gene family under different stresses, which is highly corresponded with the previous research. In most cases, all validated *ALDH* genes were upregulated in salt stress conditions, and most *ALDH* genes were significantly compared with control and *BjALDH22* was the most significant. However, the expression of most *ALDH* genes also showed an upregulation trend for drought stress, except for *BjALDH8* and *BjALDH25*, which was not significant compared with the salt stress conditions, while *BjALDH5* was downregulated. This suggests that *BjALDH* may control the process of the aldehyde metabolism by changing the expression level of the mustard *ALDH* genes, which may be more effective for salt stress than for drought stress.

In the face of various abiotic stresses, plants produce excessive aldehydes, which cause toxicity and abnormal mutations at high physiological concentrations [40], leading plants to regulate the levels of these substances in cells in response to these stresses. The members of the *ALDH* gene family are highly likely to regulate this physiological process through their gene expression products. Moreover, this study found response mechanisms of *ALDHs* to many other cis-acting elements, such as hypoxia signal response elements and cell cycle regulatory elements, implying that the *ALDH* gene family may also play an important role in these physiological activities. For example, Tola et al. [12] found that the *ALDH* gene in plants is not only deeply involved in the physiological process of dealing with abiotic stress, but it affects the amino acid metabolism, lipid metabolism, and *ALDH3F1* by participating in histone acetylation, regulating FLC gene (a key inhibitor of flowering time regulation) expression, and affects the plant flower organ development and flowering time. Moreover, it has been demonstrated *ALDH* genes were differentially expressed in soybean roots and leaves under selenium stress, and the level of gamma aminobutyric acid was regulated to reduce oxidative damage in cells under selenium stress, while maintaining redox balance in cells [41]. These studies provide some reference and guiding value for exploring the role and role of the *ALDH* gene family in various physiological processes in plants.

However, this study has some limitations. The experimental conditions were limited to controlled laboratory settings, and the stress responses of plants in actual field environments may differ. Future research should integrate field trials to validate the practical applicability and generalizability of the *BjALDH* gene family in conferring stress tolerance. While this work highlights potential linkages between *BjALDH* and key stress-responsive signaling pathways—particularly the ABA signaling pathway, ROS homeostasis, and the SOS pathway—the mechanistic interactions underlying these pathways remain to be fully elucidated. Subsequent investigations are required to dissect the molecular crosstalk and regulatory networks involving *BjALDH* under combinatorial stress scenarios. Overall, our study provides valuable insights into the functions and potential mechanisms of the *ALDH* gene family in *B. juncea* under drought and salt stress. The findings lay a theoretical foundation for future genetic improvement of stress-resistant mustard varieties and offer new perspectives for exploring the roles of the *ALDH* gene family in plant responses to abiotic stresses.

## 5. Conclusions

A total of 39 members of the mustard *ALDH* gene family were identified, distributed on 15 chromosomes. The *ALDH* genes of *B. juncea* are divided into 10 subgroups, with similar genes and protein structures within the same subgroup. There are 494 photoresponsive elements in the promoter region of the *BjALDH* family genes, which may help the plants adapt to light changes, enhance antioxidant defense, and regulate metabolic processes by regulating the expression of the *ALDH* genes. In addition, the expression of *BjALDH* was induced by salt stress and drought stress, indicating that it may be involved in the response process of mustard to salt/drought stress. Nine *ALDH* genes were upregulated significantly under salt stress, the most significant upregulation of them is *BjALDH22*, which is upregulated more than 40-fold compared with controls. *BjALDH8 BjALDH25* were upregulated significantly, and seven *ALDH* genes were not significantly upregulated under drought stress.

## Figures and Tables

**Table 1:** Primer sequence information used in this study.

Gene Name	Forward Primer (5′ 3′)	Reverse Primer (5′ 3′)
*BjALDH3*	CTCGGAAGGTTGGCCCTG	TGCAAATTCAGCCGCAGC
*BjALDH5*	GGTCGGCCACATCATCCC	CTGCTGGCTTGACCACCA
*BjALDH7*	TGTGAGGACGCCGATGTC	CACCCGCACAGTTTTGCC
*BjALDH8*	GCAAGCTGCGGGTGAAGT	GGACCATAAGGCCAGCGG
*BjALDH11*	TGGGGTGGAGTCAAACGC	GCATCCCCATGGGTCGTT
*BjALDH15*	GCCGTGAAGGAGACTGTGG	CTCCGAGCTGTGCCTTCC
*BjALDH21*	CAACTGCTGGAGCTGCCA	GATTGCTTGCTGCTGCGG
*BjALDH22*	GCCATGGAGGAGACTGTGG	TCCGAGCTGTGCCTTCCT
*BjALDH25*	GCACTGGTCTGCGGAAAC	TGGCACCGGGTAAACTGT
*GAPDHF*	TCAGTTGTTGACCTCACGGTT	CTGTCACCAACGAAGTCAGT

**Table 2:** Physicochemical properties and subcellular localization prediction of the BjALDH proteins.

Gene ID	Gene Name	Number of Amino Acids	Formula Weight/Da Molecular Weight	Isoelectric Point pI	Instability Index Instability Index	Average Hydrophobicity Gravy
BjuA01g40020S	*BjALDH1*	585	64,272.48	8.35	40.06	0.082
BjuA01g41590S	*BjALDH2*	481	53,511.23	7.56	36.24	−0.023
BjuA02g19160S	*BjALDH3*	205	22,174.78	8.27	31.46	0.219
BjuA03g04550S	*BjALDH4*	484	53,896.72	8.29	35.02	−0.035
BjuA03g21040S	*BjALDH5*	501	54,329.36	5.99	31.97	−0.085
BjuA05g18570S	*BjALDH6*	573	63,434.63	8.47	40.46	0.068
BjuA05g35930S	*BjALDH7*	666	73,865.62	8.45	42.26	0.098
BjuA06g17280S	*BjALDH8*	557	61,799.07	6.45	36.19	−0.137
BjuA06g19000S	*BjALDH9*	503	54,788.03	5.83	28.18	−0.049
BjuA06g19030S	*BjALDH10*	537	58,771.01	8.63	33.06	−0.147
BjuA07g24680S	*BjALDH11*	909	99,378.23	5.44	36.32	0.009
BjuA07g37710S	*BjALDH12*	556	59,787.01	7.07	43.29	0.088
BjuA08g01080S	*BjALDH13*	509	54,209.34	5.44	34.6	0.052
BjuA08g05370S	*BjALDH14*	482	53,035.76	8.11	38.35	0.062
BjuA08g18560S	*BjALDH15*	492	54,640.65	8.33	34.98	−0.013
BjuA08g23210S	*BjALDH16*	537	58,617.06	7.19	27.45	−0.089
BjuA10g06430S	*BjALDH17*	254	28,363.27	9.08	44.8	0.017
BjuB02g32810S	*BjALDH18*	58	5911.94	8.21	26.45	0.66
BjuB02g74420S	*BjALDH19*	484	53,958.68	8.05	36.4	−0.062
BjuB03g14960S	*BjALDH20*	126	13,802.94	4.27	34.53	0.329
BjuB03g38000S	*BjALDH21*	501	54,415.44	5.93	35.62	−0.077
BjuB03g47710S	*BjALDH22*	488	54,232.29	8.47	33.9	0.011
BjuB04g00710S	*BjALDH23*	475	52,727.58	9.34	37.01	−0.005
BjuB04g01700S	*BjALDH24*	205	22,113.56	5.73	29.67	0.074
BjuB04g08660S	*BjALDH25*	124	13,520.87	4.99	34.83	0.513
BjuB04g21940S	*BjALDH26*	523	56,972.18	7.67	26.38	−0.054
BjuB05g01010S	*BjALDH27*	133	14,099.38	8.46	42.56	0.237
BjuB05g60440S	*BjALDH28*	481	53,471.12	7.07	37.24	−0.029
BjuB06g01260S	*BjALDH29*	528	56,475.96	7.54	41.71	0.005
BjuB06g14620S	*BjALDH30*	86	9294.79	9.46	28.44	−0.206
BjuB06g16690S	*BjALDH31*	906	98,933.92	5.59	35.44	0.03
BjuB06g26910S	*BjALDH32*	482	53,166.87	8.13	40.76	0.068
BjuB06g30770S	*BjALDH33*	253	26,986.02	6.44	22.42	0.068
BjuB06g31160S	*BjALDH34*	96	10,132.99	9.69	36.5	0.493
BjuB06g47790S	*BjALDH35*	520	57,688.28	6.9	35.8	−0.171
BjuB07g01690S	*BjALDH36*	508	54,135.26	5.44	35	0.05
BjuB07g08290S	*BjALDH37*	555	60,815.45	8.01	42.87	0.018
BjuB07g14010S	*BjALDH38*	242	26,062.94	6.44	25.97	0.002
BjuB08g11910S	*BjALDH39*	538	58,501.85	8.06	32.23	−0.073

## Data Availability

All the data generated or analyzed during this study are included in the published article and its Supplementary Information Files.

## Supplementary Materials

The following supporting information can be downloaded at https://www.mdpi.com/article/10.3390/genes16050559/s1, Supplementary File S1: Detail sequence information of the B. napus *BjALDH* protein family. Supplementary File S2: ALDHs amino acid sequences in *B. juncea*, *A. thaliana* and *C. sinensis* in the phylogenetic tree.

## Abbreviations

The following abbreviations are used in this manuscript:

ALDH	Aldehyde Dehydrogenase
qRT-PCR	quantitative Real-Time Polymerase Chain Reaction
NaCl	Sodium Chloride
PBS	Phosphate Buffered Saline
DNA	Deoxyribonucleic Acid
RNA	Ribonucleic Acid
Gm.	*Glycine max*
Ca.	*Capsicum annuum*
At.	*Arabidopsis thaliana*
St.	*Solanum tuberosum*
Bj.	*Brassica juncea*
Cs.	*Camellia sinensis*
SS	salt stress
DS	drought stress
CK	control
mM	millimoles
Os.	*Oryza sativa*
FLC	Flowering Locus C
ABA	Abscisic acid
